# A Novel RPL Algorithm Based on Chaotic Genetic Algorithm

**DOI:** 10.3390/s18113647

**Published:** 2018-10-27

**Authors:** Yanan Cao, Muqing Wu

**Affiliations:** Beijing Key Laboratory of Network System Architecture and Convergence, Beijing University of Posts and Telecommunications, Beijing 100876, China; wumuqing@bupt.edu.cn

**Keywords:** chaotic genetic algorithm, objective function, RPL, routing metrics

## Abstract

RPL (routing protocol for low-power and lossy networks) is an important candidate routing algorithm for low-power and lossy network (LLN) scenarios. To solve the problems of using a single routing metric or no clearly weighting distribution theory of additive composition routing metric in existing RPL algorithms, this paper creates a novel RPL algorithm according to a chaotic genetic algorithm (RPL-CGA). First of all, we propose a composition metric which simultaneously evaluates packet queue length in a buffer, end-to-end delay, residual energy ratio of node, number of hops, and expected transmission count (ETX). Meanwhile, we propose using a chaotic genetic algorithm to determine the weighting distribution of every routing metric in the composition metric to fully evaluate candidate parents (neighbors). Then, according to the evaluation results of candidate parents, we put forward a new holistic objective function and a new method for calculating the rank values of nodes which are used to select the optimized node as the preferred parent (the next hop). Finally, theoretical analysis and a series of experimental consequences indicate that RPL-CGA is significantly superior to the typical existing relevant routing algorithms in the aspect of average end-to-end delay, average success rate, etc.

## 1. Introduction

Low-power and lossy networks (LLNs) [1,2], composed from a few dozen to thousands of nodes, are a kind of communication network which can support the following three kinds of communication modes: (1) point-to-point (traffic flow between two nodes); (2) point-to-multipoint (traffic flow from a node to multiple nodes); (3) multipoint-to-point (traffic flow from multiple nodes to a central node). The nodes and their interconnected links in LLN are constrained. That is the nodes are limited in memory capacity, processing power, and energy. Moreover, the interconnected links are characterized by lower data transmission rate, lower bandwidth, higher packet loss ratio, and worse network stability compared with other kind of wireless connected links. Furthermore, an IPv6 routing protocol for low-power and lossy networks (RPL) [3,4,5,6], proposed by Internet Engineering Task Force (IETF), is known as a dedicated routing algorithm being utilized for LLNs.

Despite the fact that RPL can support widespread applications for LLNs, it is still under improvement. In consequence, a novel algorithm of RPL based on a chaotic genetic algorithm (RPL-CGA) is proposed. RPL-CGA can significantly ameliorate the network performance of LLNs. The following 6 points are the major contributions of this paper.

(1) A composition metric is proposed, which simultaneously considers packet queue length in the buffer, end-to-end delay, residual energy ratio of nodes, number of hops, and expected transmission count (ETX) when selecting preferred parents (the next hop). These five routing metrics have a significant effect on routing decisions. Hence, to choose the best paths, all of the routing metrics mentioned above should be evaluated comprehensively.
(2) The routing algebra theoretical framework which ensures consistency, optimality, and no-loop for the newly-proposed routing metrics is analyzed.
(3) RPL-CGA uses a chaotic genetic algorithm to determine weighting factors of routing metrics in composition metrics to comprehensively evaluate candidate parents (neighbors) when selecting preferred parents. In this way, the best weighting factors allocation scheme can be obtained. Then, among many candidate parents (neighbors), the optimal preferred parent (the next hop) can be selected.
(4) A new holistic objective function is proposed. This objective function can provide a better description of the optimal routes to destination nodes, and can also provide a more comprehensive evaluation of candidate parents when selecting preferred parents.
(5) A new method for calculating the rank values of nodes is proposed. Here, rank values are used to construct the network topologies and select the preferred parent (the next hop).
(6) Simulation studies of RPL-CGA and several typical existing relevant routing algorithms are carried out in this paper. Simulation results demonstrate that RPL-CGA is superior to these typical existing relevant routing algorithms, and can obtain considerable enhancement on network performance of LLNs in the aspects of average end-to-end delay, average success rate, etc.

The rest of this paper is structured as follows: Section 2 explains RPL and the recent research works related to our problems. Section 3 introduces the new created RPL-CGA algorithm and analyzes network performances in detail. Section 4 gives the experiment consequences and analysis of RPL-CGA and another popular related algorithm. Finally, this paper is discussed and concluded with future research directions in Section 5 and Section 6.

## 2. Overview and Problems of RPL

This section gives an introduction of RPL and points out the problems with its current improvements.

### 2.1. Introduction of RPL

**Figure 1:**
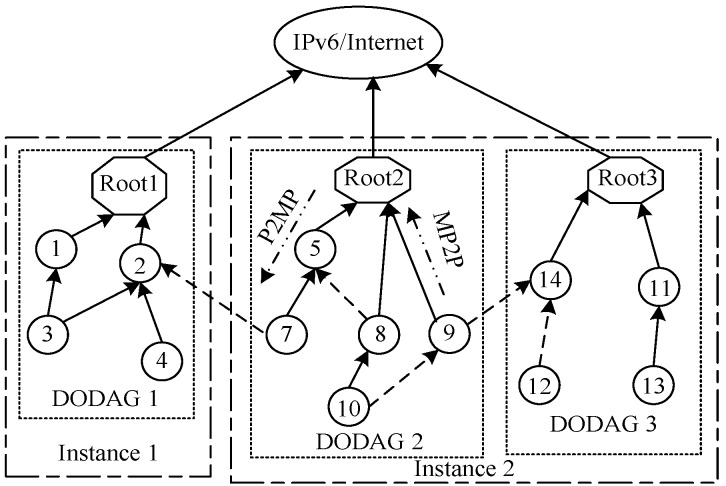
RPL Network.

RPL [3,7], standardized by IETF, is a source routing protocol. It can support widespread applications such as NAN (Neighbor Area Networks) and HAN (Home Area Networks) in smart grids [8,9], wireless sensor networks, etc. As shown in Figure 1, the RPL network can be constructed and maintained by DODAG (Destination-Oriented Directed Acyclic Graph, DODAG), which is a directed acyclic graph that each edge is oriented toward and terminating at root. DODAG can effectively prevent routing loop problems, and can be established according to ICMPv6 control messages and objective function (OF) OF 14 [10,11,12]. ICMPv6 control messages used in RPL and their corresponding functions are listed in Table 1.

OF, another important tool in RPL, is used to define the rules for network topology construction and routing decisions in LLNs, such as “the minimum ETX”. The main rules of RPL nodes defined by OF are as follows:

(1) How to get and update routing metrics information;
(2) How to calculate the rank of node (the individual position of the node relative to root in DODAG);
(3) How to choose the node’s preferred parent (the next hop node).

Furthermore, based on diverse application scenario requirements, various OFs can be designed, such as OF0 [13], ETXOF [14], MRHOF (Minimum Rank with Hysteresis OF) [15], etc.

### 2.2. Problems

At present, it has been pointed out that network performance can be improved by constructing different composite metrics. In Ref. [13], authors proposed the minimum rank rule, which means that in the candidate parents set (neighbor set), the candidate parent with the minimum rank value will be selected as the preferred parent (the next hop). But the routing metrics proposed in [16] were not considered. Moreover, the minimum rank rule does not perform any load balancing operations, which may affect the network performances to some extent. In Ref. [15], O. Gnawali and P. Levis put forward MRHOF, a kind of OF that decides routes according to minimum routing metric rules, while employing hysteresis to decrease disturbance (avoid changing the preferred parent frequently) in response to small routing metric value changes. In other words, MRHOF decides routes based on the following two rules:

(1) Node chooses the candidate parent with smallest path cost as preferred parent;
(2) If one node already selected a preferred parent but there is another candidate parent with the minimum path costing less than the selected preferred parent, then before changing its preferred parent, the node shall first compare the difference value of path cost between the minimum path cost candidate parent and the selected preferred parent. Then, if the difference value is greater than the pre-set threshold, the node chooses this minimum path cost candidate parent as its new preferred parent. Otherwise, the node still uses its current preferred parent.

This is the so called “hysteresis”. However, MRHOF cannot reflect the network situation in a timely manner due to “hysteresis” and without load balancing.

In Ref. [17], the authors put forward the hybrid routing metrics to improve the network performance. The proposed hybrid routing metric includes lexical and additive methods. In the lexical approach, the node chooses the preferred parent according to the following rules: the neighbor (candidate parent) with the maximum or minimum value of the first referential routing metric should be chosen as the preferred parent, while if there are two or more candidate parents having the same minimum or maximum value of this first referential routing metric, the second referential routing metric should be used (use the same rules as the first referential routing metric). In the additive approach, several routing metrics and their relative weighting factors are combined in an additive manner to form a composite function which is used to select the preferred parent. The weighting factors in composite function may be altered to focus on the corresponding metrics. However, the lexical way often cannot make all referential routing metrics valid. Since there are two or more candidate parents with the same minimum or maximum value of the first referential routing metric, the second referential metric takes effect; however, this circumstance—let alone the third or other subsequent routing metrics—rarely occurs.. 

Furthermore, the additive way has no definite weighting factors distribution theory analysis and basis. The determination of the weighting factor of each metric is mainly based on experts’ personal experiences. It is too subjective and cannot alter the corresponding weighting factors in a timely manner according to the changes of the network. In Ref. [18,19,20,21], the authors use the additive method to composite several routing metrics to improve the network performance. Different simulation results with different weighting factor distribution schemes are given. However, similar to [15], all of them have no definite weighting factors distribution theory analysis and basis. The weighting factor of each metric is determined based on subjective experiences and experimental results instead of rigorous weighting distribution theory.

In conclusion, RPL and its improvements based on composition metrics in the additive way have the following problems:

(1) Packet queue length in buffer, end-to-end delay, residual energy ratio of nodes, number of hops, and expected transmission count (ETX) all have an important influence on routing selection. However, the recent improvements of RPL only consider two or three routing metrics. So, the optimal routes are hard to select, and the network performance is affected to some extent.
(2) It is too subjective that the weighting factor of each routing metric is decided by the personal experiences of experts. There is no definite analysis and basis about weighting factors distribution theory of each routing metric used in composition metric.
(3) The weighting factors of routing metrics cannot be dynamically adjusted according to network changes. Therefore, the network performances are affected to a certain extent.

To solve these problems, RPL-CGA, a novel improved algorithm of RPL based on a chaotic genetic algorithm is presented. By adopting the newly-proposed chaotic genetic algorithm to optimize the weighting factor of each routing metric in composition metric to assess candidate parents (neighbors) all sidedly, RPL-CGA will choose the optimum candidate parent as the preferred parent (the next hop) and achieve significant improvement on network performance of LLNs in the aspect of average end-to-end delay, average success rate, etc.

## 3. RPL-CGA

In this section, RPL-CGA, the newly-proposed algorithm will be introduced at length.

### 3.1. Outlines of RPL-CGA

The outlines of RPL-CGA are as follows:

(1) Analyzing CGA.
(2) Analyzing the requirements that RPL routing metrics should meet.
(3) Proposing which routing metrics should be considered in RPL-CGA.
(4) Proposing novel composition metric and objective function.
(5) Using the chaotic genetic algorithm to optimize the weighting factor of each routing metric in the composition metric.
(6) Calculating rank values of nodes according to the newly-proposed objective function.
(7) Choosing the preferred parents based on rank values of nodes.

In what follows, the details of RPL-CGA are described according to the abovementioned seven sections.

### 3.2. CGA

A genetic algorithm is a kind of heuristic algorithm. It simulates the evolution process of biological populations in nature, and mainly includes genetic, crossover, and mutation operations. It can directly operate on the object, has better global optimization ability, can automatically obtain and guide the optimized search space, adaptively adjust the search direction, and does not need to determine the relevant search rules. It can be widely used in function optimization, image processing, machine learning, etc. However, the genetic algorithm has some problems such as premature convergence and local optimum.

Chaos appears to be irregular and similar to random, but it has a delicate internal structure. A chaos optimization algorithm has the characteristics of avoiding local optimization, randomness, and ergodicity in search processes. The definition of chaos is as follows:

**Definition** **1.** *The continuous self-mapping f on [a, b] is chaotic, if the following conditions are satisfied:*

(1) The period of the periodic point of f has no upper bound.
(2) Existing uncountable subset S⊂[a, b], there is no periodic point in S, and the following conditions are met:
  *①*  ∀x,y∈S, $\underset{n\to \infty }{\lim}\inf\left|f^{n}(x)-f^{n}(y)\right|=0$;
  *②*  ∀x,y∈S, x≠y, $\underset{n\to \infty }{\lim}\sup\left|f^{n}(x)-f^{n}(y)\right|>0$;
  *③*  ∀x∈S, *y is any periodic point of f*, $\underset{n\to \infty }{\lim}\sup\left|f^{n}(x)-f^{n}(y)\right|>0$.

Based on the characteristics of chaotic optimization and genetic algorithm, a chaotic genetic algorithm (CGA) is proposed in this paper. The basic idea is to introduce the chaotic mechanism into genetic algorithm, and to combine the genetic algorithm and chaotic optimization algorithm to complement each other. The overall process of CGA is basically consistent with the genetic algorithm, such as the generation of the initial population, fitness calculation, selection, crossover, mutation, replication, etc. Moreover, CGA improves the selection of control parameters and the initial value of initial population, and reduces the randomness of the genetic algorithm. Meanwhile, convergence and local optimization of the genetic algorithm can be effectively improved through chaotic selection, crossover, mutation, etc. In other words, CGA can enlarge the search sample space due to the ergodicity of chaotic algorithm, can find the global optimal solution due to the global search of genetic algorithm, and can avoid local optimization due to the initial sensitivity of chaotic optimization algorithm. Meanwhile, the population size has little effect on searching for the global optimal solution. Therefore, CGA is one of the best candidates to solve optimization problems. So RPL-CGA is proposed in this paper to search for the optimal solution of routing metrics’ weighting factors in composition metric to comprehensively evaluate candidate parents (neighbors) when selecting preferred parents. In this way, the best weighting factors allocation scheme can be obtained. Then among many candidate parents (neighbors), the optimal preferred parent (the next hop) can be selected and achieve significant improvement on network performance of LLNs in the aspect of average end-to-end delay, average success rate, etc.

### 3.3. Requirements for RPL-CGA Routing Metrics

To optimize the network performance, the employed routing protocol should abide by the properties of consistency, optimality, and no-loop. Consistency means that if packets from node *v*_1_ to *v_n_* are delivered through the path *P*(*v*_1_, *v_n_*) = (*v*_1_, *v*_2_,⋯, *v_n_*), then other nodes on path ***P*** must take the same decisions. For example, packets form *v*_2_ to *v_n_* should select path *P*(*v*_2_, *v_n_*) = (*v*_2_, *v*_3_,⋯, *v_n_*), and the same for *v*_3_, *v*_4_,⋯, *v_n_*_−1_. Optimality means that the routing protocol should transmit packets through the lightest path (minimal overhead, delay, or other metrics) or optimal routes among nodes. No-loop means that for every path from source to destination, any two nodes on the path are different.

According to Ref. [18,22] isotonicity and monotonicity of routing metrics are enough to guarantee that the routing protocol can produce consistency, optimality, and no-loop path during route computation period. So in the following paragraphs, isotonicity and monotonicity of routing metrics are given in detail.

First, this paper models the RPL network as a DAG (Directed Acyclic Graphic, DAG) *G*(*V*, *E*), where *V* and *E* indicate the finite set of nodes and links between nodes respectively. One path *P* from node *s* to *d* can be expressed as *P*(*v_s_*, *v_d_*) = (*v_s_*, *v_s+_*_1_, *v_s+_*_2_,⋯, *v_d_*), where $P(v_{s},v_{d})\in \Omega$ (the finite set of all the paths). Then, suppose that $F(P(v_{s},v_{d})\in \Omega )$ is the mapping function which is used to calculate the metric value (e.g., hop count, ETX, residual energy, end-to-end delay, and packet queue length in buffer) of the path between nodes *s* and *d*. Each path between source and destination has a value that can be ordered to select the best path. The isotonicity and monotonicity of the routing metrics are explained as follows.

(1) Monotonicity

If the metric value of a path or link does not decrease when suffixed or prefixed by another link or path, the metric is monotonic. The mathematical expression is as follows:

$$\begin{array}{l} F(P(v_{a},v_{b}))\leq F(P(v_{a},v_{b})⊕P(v_{b},v_{c})),F(P(v_{a},v_{b}))\leq F(P(v_{c},v_{a})⊕P(v_{a},v_{b})),\\ \{\forall P(v_{a},v_{b}),P(v_{b},v_{c}),P(v_{c},v_{a})\in \Omega \}. \end{array}$$

(2) Isotonicity

If the order relation of any two links or paths is invariability when prefixed or suffixed by a third link or path, the metric is isotonic. The mathematical expression is as follows:

$$\begin{array}{l} F(P(v_{a},v_{b}))\leq F(P(v_{c},v_{d}))\Rightarrow \{\begin{array}{c} F(P(v_{a},v_{b})⊕P(v_{e},v_{f}))\leq F(P(v_{c},v_{d})⊕P(v_{e},v_{f}))\\ F(P(v_{g},v_{h})⊕P(v_{a},v_{b}))\leq F(P(v_{g},v_{h})⊕P(v_{c},v_{d})) \end{array}\\ \{\forall P(v_{a},v_{b}),P(v_{c},v_{d}),P(v_{e},v_{f}),P(v_{g},v_{h})\in \Omega \}. \end{array}$$

### 3.4. Routing Metrics Considered in RPL-CGA

This paper proposes five representative routing metrics to be used to create a composition metric which will be used in RPL-CGA. Suppose that the node *c* has *n* candidate parents.

(1) Queue length (QL)

QL is the packet queue length of nodes. As shown in Equation (1), *QL*_max_ represents the maximum queue length of all candidate parents. *QL*(*i*) (i=1,2,⋯,n) is the packet queue length in the buffer of candidate parent *i*. It is more inclined to choose the candidate parent (neighbor) with the lowest packet queue length as the preferred parent. Therefore, QL can be used to balance the network load and select the optimum preferred parent (the next hop).

(1) $$g_{1}(i)=\frac{QL(i)}{QL_{\max}}$$

(2) End-to-End delay (EED)

EED is the summation of all link delay. As shown in Equation (2), *EED_max_* represents the maximum EED of the path from this node via a candidate parent to root. Let *EED*(*i*) (i=1,2,⋯,n) indicates the end-to-end delay of one route from a node via its candidate parent *i* to root. Here, *EED*(*i*) includes the following two sections: (a) the end-to-end delay of the link between node and its candidate parent *i*; (b) the end-to-end delay in DIO broadcast by candidate parent *i*. In addition, if node via candidate parent *e* has the minimum *EED*(*e*) among all candidate parents, then *e* will be chosen as the preferred parent.

(2) $$g_{2}(i)=\frac{EED(i)}{EED_{\max}}$$

(3) Residual energy ratio (RER)

As shown in Equation (3), *g*_3_(*i*) (i=1,2,⋯,n) represents the residual energy ratio of a candidate parent *i*. *E_initial_*(*i*) and *E_current_*(*i*) represent the maximum initial energy and the current energy of candidate parent *i* respectively. Therefore, it is more inclined to choose the candidate parent (neighbor) with the maximum residual energy as the preferred parent to prolong the network lifetime. Although the remaining energy is a concave metric, some transformations of remaining energy are done to make the derived metric (*g*_3_(*i*) (i=1,2,⋯,n)) coincide with other metrics in metric range, operator, order relation, isotonicity, monotonicity etc.

(3) $$g_{3}(i)=1-\frac{E_{current}(i)}{E_{initial}(i)}$$

(4) Hop number count (HC)

Hop count is the number of hops between candidate parent and root. As shown in Equation (4), *HC_max_* represents the maximum hop count between a candidate parents and root. *HC*(*i*) (*i* = 1, 2, …, *n*) represents the number of hops between candidate parent *i* and root. In addition, if the candidate parent *f* has the minimum *HC*(*f*) among all candidate parents, then *f* will be chosen as the preferred parent.

(4) $$g_{4}(i)=\frac{HC(i)}{HC_{\max}}$$

(5) ETX

ETX (Expected Number of Retransmissions, ETX) represents the expected number of transmissions or retransmissions needed to successfully transmit and acknowledge one packet on the link. As shown in Equation (5), *ETX_max_* represents the maximum ETX of the path from this node through a candidate parent to root. *ETX*(*i*) (i=1,2,⋯,n) indicates the ETX from one node via candidate parent *i* to root. Here, *ETX*(*i*) includes the following two sections: (a) the ETX of the link between node and its candidate parent *i*; (b) the ETX in DIO (the DIO message is broadcast by candidate parent *i*). In addition, if node via candidate parent *l* to root has the smallest *ETX*(*l*) among all candidate parents, then *l* will be chosen as the preferred parent.

(5) $$g_{5}(i)=\frac{ETX(i)}{ETX_{\max}}$$

In general, every routing metric has a significant impact on routing decisions. Hence, for the sake of choosing the best routes, RPL-CGA should assess all the above proposed routing metrics. Furthermore, the abovementioned five representative routing metrics can easily be proven to be isotonicity and monotonicity. Therefore, all of them are suitable candidate routing metrics to create a composition metric.

### 3.5. Proposing Composition Metric and Objective Function

As shown in Equation (6), the abovementioned five representative routing metrics can be united to create a composition metric (CM) in an additive way. *a*_1_, *a*_2_, *a*_3_, *a*_4_, *a*_5_ are weighting factors to adjust the impact of each routing metric. *n* indicates the number of candidate parents.

(6) $$\begin{array}{l} {F(i)}_{CM}=\sum_{j=1}^{5}a_{j}g_{j}(i),\;(i=1,2,\cdot \cdot \cdot ,n)\\ s.t.\{\begin{array}{c} \sum_{j=1}^{5}a_{j}=1\\ 0\leq a_{j}\leq 1 \end{array} \end{array}$$

The newly-proposed composition metric also meets monotonicity and isotonicity. The proof is as follows:

First, suppose there exists three paths *P*_1_ = *P*(*v_s_*, *v_d_*)=(*v_s_*, *v_s+_*_1_, *v_s+_*_2_,⋯, *v_d_*), *P*_2_ = *P*(*v*′*_s_*, *v*′*_d_*)=(*v*′*_s_*, *v*′*_s+_*_1_, *v*′*_s+_*_2_,⋯, *v*′*_d_*), *P*_3_=*P*(*v*″*_s_*, *v*″*_d_*)=(*v*″*_s_*, *v*″*_s+_*_1_, *v*″*_s+_*_2_,⋯, *v*″*_d_*). Then, the composition metric values of these three paths are as follows:

$$F(P_{1}){}_{CM}=F((v_{s},v_{s+1}){}_{CM}⊕(v_{s+1},v_{s+2}){}_{CM}⊕\cdots ⊕(v_{d-1},v_{d})){}_{CM}=F(v_{s}){}_{CM}+F(v_{s+1}){}_{CM}+\cdots +F(v_{d}){}_{CM}\geq 0\;F(P_{2}){}_{CM}=F(({v^{\prime }}_{s},{v^{\prime }}_{s+1}){}_{CM}⊕({v^{\prime }}_{s+1},{v^{\prime }}_{s+2}){}_{CM}⊕\cdots ⊕({v^{\prime }}_{d-1},{v^{\prime }}_{d})){}_{CM}=F({v^{\prime }}_{s}){}_{CM}+F({v^{\prime }}_{s+1}){}_{CM}+\cdots +F({v^{\prime }}_{d}){}_{CM}\geq 0\;\;F(P_{1}){}_{CM}=F(({v^{\prime \prime }}_{s},{v^{\prime \prime }}_{s+1}){}_{CM}⊕({v^{\prime \prime }}_{s+1},{v^{\prime \prime }}_{s+2}){}_{CM}⊕\cdots ⊕({v^{\prime \prime }}_{d-1},{v^{\prime \prime }}_{d})){}_{CM}=F({v^{\prime \prime }}_{s}){}_{CM}+F({v^{\prime \prime }}_{s+1}){}_{CM}+\cdots +F({v^{\prime \prime }}_{d}){}_{CM}\geq 0$$

(1)  Monotonicity

The composition metric value of every path or link is always greater than or equal to 0; therefore, regardless of being prefixed or suffixed by another link or path such as *P*_3_, $F(P_{1}){}_{CM}\leq F(P_{1}){}_{CM}+F(P_{3}){}_{CM}$ can easily be proven, so the newly-proposed composition metric meets monotonicity.

(2) Isotonicity

The composition metric value of every path or link is always greater than or equal to 0. If $F(P_{1}){}_{CM}\leq F(P_{2}){}_{CM}$, then, regardless of being prefixed or suffixed by another link or path such as *P*_3_, $F(P_{1}){}_{CM}+F(P_{3}){}_{CM}\leq F(P_{2}){}_{CM}+F(P_{3}){}_{CM}$ can easily be proven, so the newly-proposed composition metric meets isotonicity.

Therefore, the newly-proposed composition metric meets monotonicity and isotonicity.

As shown in Equation (7), the newly-proposed RPL-CGA objective function can be defined as the minimum function of the proposed composition metric.

(7) $$\begin{array}{l} OF_{RPL-CGA}={\min F(i)}_{CM}=\min\;(\sum_{j=1}^{5}a_{j}g_{j}(i)),\;(i=1,2,\cdot \cdot \cdot ,n)\\ s.t.\{\begin{array}{c} \sum_{j=1}^{5}a_{j}=1\\ 0\leq a_{j}\leq 1 \end{array} \end{array}$$

Meanwhile, it can be seen that each weighting factor determines the importance of the corresponding routing metric in the composition metric, which, in turn, affects the choice of preferred parents. Therefore, the optimization of routing metrics weighting factors becomes a crucial issue. However, the existing improvements of RPL in an additive manner determine the weighting factors of routing metrics according to experts’ personal experiences. It is too subjective and inefficient, and cannot alter weighting factors in a timely manner according to the changes of the network to satisfy the QoS requirements well. To address these problems, RPL-CGA uses a chaotic genetic algorithm [23,24] to optimize the weighting factors of routing metrics in the composition metric to evaluate candidate parents comprehensively when selecting preferred parents. In this way, RPL-CGA can easily choose the optimum candidate parent (neighbor) as the preferred parent (the next hop), and achieves significant improvement on the network performance of LLNs in the aspect of average end-to-end delay, average success rate, etc.

### 3.6. Optimizing Weighting Factors of Routing Metrics

Supposing that there are *n* nodes in the candidate parent set, and RPL-CGA considers *m* = 5 routing metrics (QL, EED, RER, HC, and ETX). Let the value of the *j*-th (j=1,2,⋯,m) metric of the *i*-th candidate parent (i=1,2,⋯,n) be expressed as *x_ij_* (i=1,2,⋯,n; j=1,2,⋯,m). Then, the sample set of each metric value at each candidate parent is $\left\{x_{ij}|i=1,2,\cdots ,n;j=1,2,\cdots ,m\right\}$. It can be written as a matrix named as initial judgment matrix **X**.

(8) $$X=\left[\begin{array}{ccccc} x_{11} & x_{12} & x_{13} & \cdot \cdot \cdot & x_{1m}\\ x_{21} & x_{22} & x_{23} & \cdot \cdot \cdot & x_{2m}\\ \cdot \cdot \cdot & \cdot \cdot \cdot & \cdot \cdot \cdot & \cdot \cdot \cdot & \cdot \cdot \cdot \\ x_{n1} & x_{n2} & x_{n3} & \cdot \cdot \cdot & x_{nm} \end{array}\right]$$

After that, the weighting factor of the *j*-th metric is set as *a_j_* (j=1,2,⋯,m), and *F*(*i*) (i=1,2,⋯,n), and the comprehensive evaluate function of candidate parents can be obtained by weighted summation of metric weighting factors and metric values *x_ij_*, as shown in Equation (9).

(9) $$F(i)=\sum_{j=1}^{m}a_{j}x_{ij},\;(i=1,2,\cdot \cdot \cdot ,n;j=1,2,\cdot \cdot \cdot ,m)$$

Therefore, through the appropriate weighting factor allocation of each metric ($a=(a_{1},a_{2},\cdots ,a_{m})$), all the candidate parents comprehensive evaluate values *F*(*i*) (i=1,2,⋯,n) can be obtained. Then node *i* with a minimum value of *F*(*i*) will be selected as the preferred parent. Here, $a=(a_{1},a_{2},\cdots ,a_{m})$, and the unit vector and weighting factor of each metric should meet the following constraint conditions:

(10) $$s.t.\{\begin{array}{c} 0\leq a_{j}\leq 1\\ \sum_{j=1}^{m}a_{j}=1 \end{array}$$

It can be seen that the selection of the preferred parent becomes a problem of finding the optimal solution under constraint conditions. That is, when the initial decision matrix is obtained, the weighting factor (*a_j_*) of every routing metric can be obtained through a multi-attribute optimization algorithm. In this way the comprehensive evaluating function *F*(*i*) of every candidate parent is obtained. Finally, the candidate parent with the lowest *F*(*i*) can be chosen as the preferred parent. For this purpose, this paper proposes the use of a chaotic genetic algorithm (RPL-CGA) to determine the weighting factor of each metric in the composition metric. Then, the preferred parents can be selected through comprehensively evaluating nodes in candidate parent set.

In RPL-CGA, the initial population genes are produced through a chaotic system, and every population individual containing *m* genes is a kind of possible metric weighting factors distribution scheme ($a=(a_{1},\;a_{2},\cdot \cdot \cdot ,\;a_{m})$). Then, the populations are improved via superior individual choice, cross, mutation, chaos perturbation, and other steps. When certain conditions are met, the algorithm ends. At this point, the population individual with the maximum fitness function value is the final weighting factors allocation scheme of routing metrics. The concrete realization processes of RPL-CGA are as follows.

Step 1: initialization

Determining relevant parameters used in RPL-CGA, such as population size *w*, cross probability *P_c_*, mutation probability *P_m_*, the maximum number of iteration *k*, etc.

Step 2: constructing fitness function

A fitness function can be used to assess the quality of population individuals. A better quality of population individuals shows greater performance and the greater probability of these individuals existing among the next generation. For the sake of ensuring the superiority of individuals, the selection of individuals is based on fitness function values. That is, individuals with greater fitness function values have a greater probability of existing among the next generation.

In RPL-CGA, the selection of preferred parents is based on the comprehensive evaluation function *F*(*i*). In contrast, the key issue of determining *F*(*i*) is determining weighting factor of each metric ($a=(a_{1},\;a_{2},\cdot \cdot \cdot ,\;a_{m})$). To this end, the fitness function can be defined as shown in Equation (11).

(11) $$Fitness(a)=\frac{1}{F(i)+1},(i=1,2,\cdot \cdot \cdot ,n)$$

(12) $$\max Fitness(a),\;s.t.\;\sum_{j-1}^{m}a_{j}=1,0\leq a_{j}\leq 1$$

It can be seen that the fitness function *Fitness*(*a*) only changes with the weighting factor variables. Therefore, as shown in Equation (12), the weighting factors can be evaluated through calculating the maximum value of the fitness function under constraint conditions. Then, the population individual corresponding to maximum *Fitness*(*a*) is the final weighting factor allocation plan of each routing metric. Therefore, this is a kind of complicated nonlinear optimal solution problem. To this end, we proposed the chaotic genetic algorithm to solve this problem.

Step 3: generating initial population by chaotic system

The chaotic system [25] is characterized by sensitive to initial values, generating better randomness sequence, traversing all state points in chaotic region, long term unpredictable etc. Therefore, this paper uses the representative logistic map [26] to produce initial population genes and chaos perturbation vectors. The system state equation of the chaotic system is shown in Equation (13).

(13) $$\tau_{z+1}=\eta \tau_{z}(1-\tau_{z}),z=0,\;1,\;2,\cdot \cdot \cdot ,\;0<\tau_{0}<1,0\leq \eta \leq 4$$

In Equation (13), η is a control parameter. When η>3.57 and τ≠0.25,0.5,0.75, the system goes into a chaotic state, and the iteration results are similar to random numbers [0, 1]. In this paper, τ=4.

RPL-CGA selects *w* population individuals and assigns *m* genes to each population individual. That is, RPL-CGA chooses w∗m population genes from a chaotic sequence produced by the chaotic system. The individual gene corresponds to the weighting factor of each routing metric. So the selection of individual genes should satisfy the constraint conditions shown in Equation (10). Then, the initial population can be recorded as $\left\{h_{i}{}_{j}|\left(i=1,2,\cdot \cdot \cdot ,w;j=1,2,\cdot \cdot \cdot ,m\right)\right\}$, and *h_i_* is a probable weighting factors distribution method of metrics ($a=(a_{1},\;a_{2},\cdot \cdot \cdot ,\;a_{m})$).

Step 4: selecting superiority individuals

RPL-CGA computes fitness function values for every **h_i_** (population individual) based on Equation (11). Then, the larger the value of *Fitness*(*h_i_*), the closer **h_i_** is to the optimal solution, and also, the better to select $h_{i}=(h_{i1},h_{i2},\cdot \cdot \cdot ,h_{im})$ as weighting factors allocation scheme. That is, RPL-CGA calculates the fitness function value *Fitness*(*h_i_*) of each population individual, arranges **h_i_** in descending order based on the fitness function values, selects the first 15% of them as superior individuals, and brings these superior individuals to the next generation population directly (no crossover, mutation, and other operations).

Step 5: generating the next generation population

The rest of 85% population individuals of the next generation are generated via crossover and mutation of the genetic algorithm.

Step 6: adding chaotic perturbation

Adding chaotic perturbation to population individuals can make RPL-CGA reduce evolution algebra, avoid local convergence, and find the best solution as quickly as possible. Moreover, RPL-CGA only applies chaotic perturbation to the 85% individuals with lower fitness function values according to Equation (14), which will shrink the search range and improve optimization efficiency.

(14) $$h^{\prime }=(1-\alpha )h+\alpha h^{\prime \prime }$$

**Figure 2:**
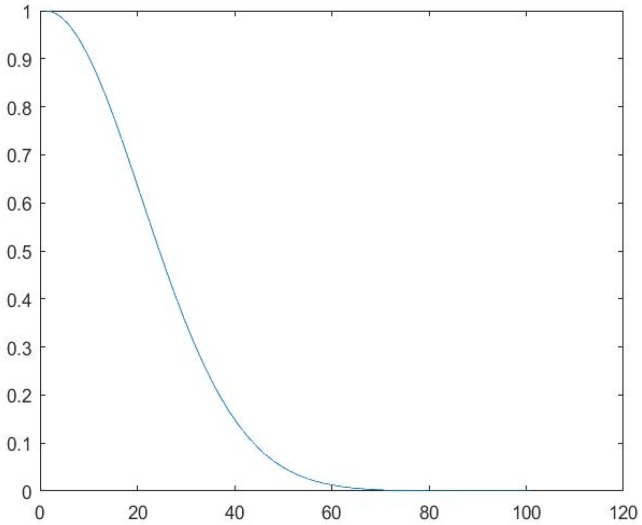
*α* curve.

In Equation (14), *h* is the current 85% population individuals generated in step 5. $h^{\prime \prime }$ is *m*-dimension chaotic perturbation vector chosen from the chaotic series. $h^{\prime }$ is the newly-generated population individuals after adding chaotic disturbance. α∈[0,1], the adjustment factor, is determined by Equation (15); the corresponding graph is shown in Figure 2.

(15) α={exp[−k22δ2], 2≤k≤40(2k−1)k2, k>40

In Equation (15), *k* is the number of iteration and δ=20. Through Equation (15), *h* changes greatly in the beginning search phase, which needs a bigger *α* value. With the search going on, *h* approaches the optimum solution gradually; so, for the sake of searching within a diminutive area close to the optimum solution, a smaller *α* is required.

Step 7: judging termination conditions

RPL-CGA stops operating if it meets Equation (16) or the iteration number reaches 100. Otherwise, it goes to step 4.

(16) $$|\bar{Fitness(a)}-Fitness{\left(a\right)}_{\max}|\leq \varepsilon$$

In Equation (16), $Fitness{\left(a\right)}_{\max}$ and $\bar{Fitness(a)}$ indicate the maximum value of fitness function and the average value of all the fitness functions respectively. $\varepsilon =10^{-5}$(a small positive number).

Step 8: preferred parents selection

After getting the optimization solution $h_{sj}=(h_{s1},h_{s2},\cdot \cdot \cdot ,h_{sm})$ through the abovementioned chaotic genetic algorithm, RPL-CGA lets $a=(a_{1},\;a_{2},\cdot \cdot \cdot ,a_{m})=h_{sj}$, brings **a** into Equation (11), and gets the fitness function values of each candidate parent. Then, the candidate parent with the maximum fitness function values will be chosen as the preferred parent, i.e., it brings **a** into Equation (9) and gets the comprehensive evaluation function values *F*(*i*) (i=1,2,⋯,n) of each candidate parent. Then, the node with the minimum comprehensive evaluation function value among all the candidate parents will be chosen as the preferred parent.

### 3.7. Calculating Rank of Nodes

The rank value of the node is calculated depending on OF. It represents the position of the node relative to root in DODAG. To prevent loops, rank values of nodes monotonically increase in the Down direction (the direction from roots towards leaf nodes) and monotonically decrease in the Up direction (the direction from leaf nodes towards roots). In Ref. [27], authors proposed a method for calculating the rank values of non-root node. Suppose that the rank value of root is 1.0; then, the rank value of c (non-root) can be calculated based on Equation (17) (the used routing metric is ETX).

(17) R(c)=128×ETX(c)+R(parent)

Here, *R*(*parent*) represents parent’s rank. *ETX*(*c*), calculated according to Equation (18), indicates the ETX of link from *c* to this parent.

(18) $$ETX(c)=ETX{\left(c\right)}_{old}\times 0.9+ETX{\left(c\right)}_{new}\times 0.1$$

In Equation (18), *ETX*(*c*)*_old_* indicates the former value, and *ETX*(*c*)*_new_* is computed through $ETX=1/D_{f}*D_{r}$. Here, *D*_f_ is the probability that a packet received by neighbor. *D_r_* is the probability that an acknowledgment packet is successfully received.

Moreover, in the set candidate parents, the candidate parent with the lowest value of Equation (17) should be chosen as the preferred parent.

In RPL-CGA, we proposed a new method to calculate the rank values of non-root nodes. Suppose that the rank value of root is 1.0. Then the rank value of c (non-root) can be calculated according to Equation (19).

(19) R(c)=R(parent)+(F(i)+1)

In Equation (19), *R*(*parent*) represents the rank of parent, and *F*(*i*) indicates the comprehensive evaluation function value of the link from c to this parent.

Then, in the candidate parents set, the one with the lowest value of Equation (19) should be chosen as the preferred parent.

### 3.8. Selecting Preferred Parents

Based on Section 3.6, in the candidate parents set, the one with the lowest value of Equation (19) will be chosen as the preferred parent (the next hop), except for in the following situations:

(1) According to Equation (19), if the value of current preferred parent is greater than the value of one candidate parent, but the difference between them is less than the preferred parent change threshold, then the current preferred parent will not be changed.
(2) If the calculated value of Equation (19) is greater than 100 or less than 1, then the corresponding candidate parent must be removed from the candidate parent set.
(3) If several candidate parents have the same minimum calculated values of Equation (19), then an additional metric named as NSA (Node State Attribute, NSA) will be considered to choose the next hop among these several candidate parents.
(4) According to Equation (19), if the value of the current preferred parent is equal to the minimum value, and there are several candidate parents also with the minimum value, then the node still uses its current preferred parent.
(5) If node *c* only has one candidate parent, then *c* should wait for some time to receive DIO messages broadcast by other nodes to determine whether there are other nodes that will become its candidate parents. After that, if *c* has two or more candidate parents, *c* selects a preferred parent through RPL-CGA algorithm. Otherwise, if c still has one candidate parent, it directly selects only one candidate parent as its preferred parent without executing the RPL-CGA algorithm.

## 4. Performance Evaluation

To demonstrate that RPL-CGA can quantitatively improve LLN performance, we conducted extensive simulation experiments through OPNET to demonstrate that RPL-CGA is superior to existing related algorithms such as ETXOF (a representative algorithm of RPL), 0.8 × ETX + 0.2 × RER and 0.6 × HC + 0.4 × RER (0.8 × ETX + 0.2 × RER and 0.6 × HC + 0.4 × RER, the famous additive manners, can get optimal performance [15]).

### 4.1. Statistical Experimetal Indicators

There are several experimental indicators studied in this paper.

(1) Average success rate

Average success rate is the proportion of successfully-received packets to the total number of sent packets, as illustrated in Equation (20).

(20) $$S=\frac{N_{r}}{N_{s}}$$

Here,$N_{r}$ is the sum of packets successfully received. $N_{s}$ is the sum of sending packets.

(2) Average end-to-end delay

Average end-to-end delay represents the average undergoing time from a data packet being sent to its arrival at the destination node, as illustrated in Equation (21).

(21) $$D=\frac{\sum t_{j}}{N_{r}}$$

Here, $t_{i}$ indicates the average undergoing time from the *j*-th data packet being sent to its arrival at the destination node. $N_{r}$ is the sum of packets which were successfully received.

(3) Network lifetime

The average residual energy of nodes or average number of dead nodes in network can clearly indicate the length of network lifetime.

(4) Average hop count

Average hop count indicates the average number of hops from source to destination.

(5) Average frequencies of preferred parent changing

Average frequencies of the preferred parent changing indicate the stability of the network’s topology. Low frequencies of preferred parent changing can change network topology little, but the optimal route selection is affected. The opposite is true for high frequencies of preferred parent changing. Therefore, the appropriate frequencies of preferred parent changing should be used.

(6) Weighting factors (*a*_1_, *a*_2_, *a*_3_, *a*_4_, *a*_5_)

The relationship between weighting factors (*a*_1_, *a*_2_, *a*_3_, *a*_4_, *a*_5_) and simulation time illustrates the changes of weighting factors of these five routing metrics with the simulation running. That is, RPL-CGA can dynamically adjust the values of *a*_1_, *a*_2_, *a*_3_, *a*_4_, and *a*_5_ according to network changes, choose the optimum parents as preferred parents, and improve network performance effectively.

### 4.2. Statistical Experimetal Indicators

We conduct extensive simulation experiments under different conditions. The experiment parameters and some related experiment rules are illustrated in Table 2.

*E*(*y*,*d*), listed in Table 1, can be computed according to Equation (22) [28].

(22) $$E(y,d)=\{\begin{array}{c} 2E_{elec}\times y+\varepsilon_{amp}\times y\times d^{2},\;d<d_{0}\\ 2E_{elec}\times y+\varepsilon_{fs}\times y\times d^{4},\;d>d_{0} \end{array}$$

In Equation (22), *E_elec_* represents the energy loss for relaying unit bit message. *ε_amp_* and *ε_fs_* are the energy needed for each unit bit message sent by the amplifier in these two circumstances, respectively. Table 3 shows the values of these parameters.

### 4.3. Results and Discuss

(1) Success rate

**Figure 3:**
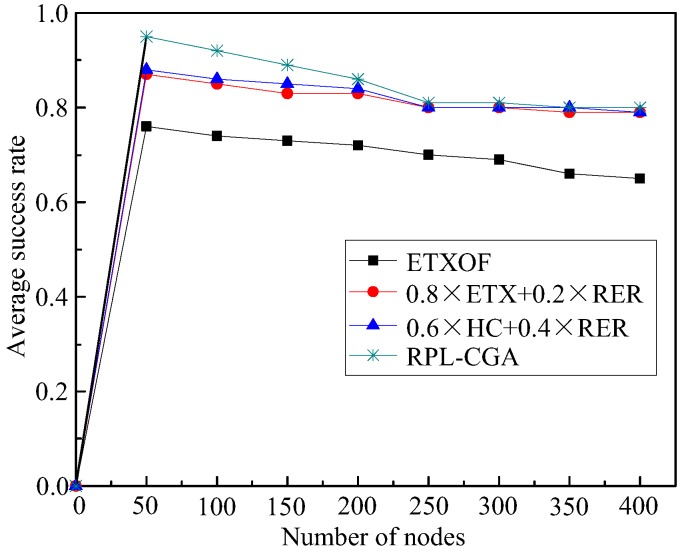
Average success rate.

Figure 3 illustrates the success rate of RPL-CGA, ETXOF, 0.8 × ETX + 0.2 × RER, and 0.6 × HC + 0.4 × RER under several different node number circumstances. It can be seen that under low node number circumstances, the success rate is high. In contrast, the average success rate is low. Furthermore, in the case of various node densities, the average success rates of RPL-CGA are all much higher than that of ETXOF, 0.8 × ETX + 0.2 × RER, and 0.6 × HC + 0.4 × RER, because ETXOF merely evaluates ETX during network topology construction and the preferred parent selection period. In that way, the selected preferred parent may encounter higher QL, HC, or EED, or lower RER. 0.8 × ETX + 0.2 × RER and 0.6 × HC + 0.4 × RER do not consider QL and EED during network topology construction and the preferred parent selection period. So, the selected preferred parent may encounter higher QL or EED. Meanwhile, it is too subjective that every weighting factor is determined by expert personal experience. Moreover, the weighting factors cannot dynamically adjust according to network changes. However, RPL-CGA can consider QL, EED, NE, HC, and ETX at the same time, determine respective weighting factors according to on chaotic genetic algorithm, and select the node with the lowest value of Equation (19) as the next hop (preferred parent). In this way, RPL-CGA can improve the success rate remarkably.

(2) Average end-to-end delay

**Figure 4:**
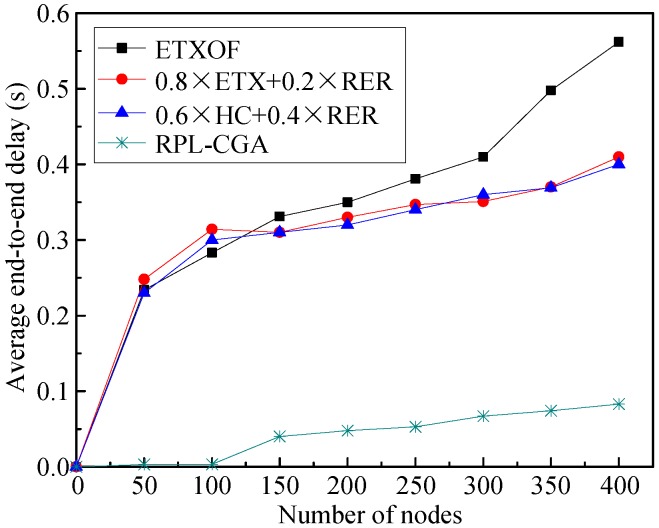
Average end-to-end delay.

Figure 4 shows the average end-to-end delays of RPL-CGA, ETXOF, 0.8 × ETX + 0.2 × RER, and 0.6 × HC + 0.4 × RER under several different node number circumstances. It can be seen that with low node numbers, the average end-to-end delay is small. But the average end-to-end delay increases as the number of nodes in the network increases. Meanwhile, the average end-to-end delay of ETXOF, 0.8 × ETX + 0.2 × RER, and 0.6 × HC + 0.4 × RER are all much higher than that of RPL-CGA at different node numbers. It is clear that RPL-CGA can effectively enhance the real-time performance of LLNs.

(3) Network lifetime

**Figure 5:**
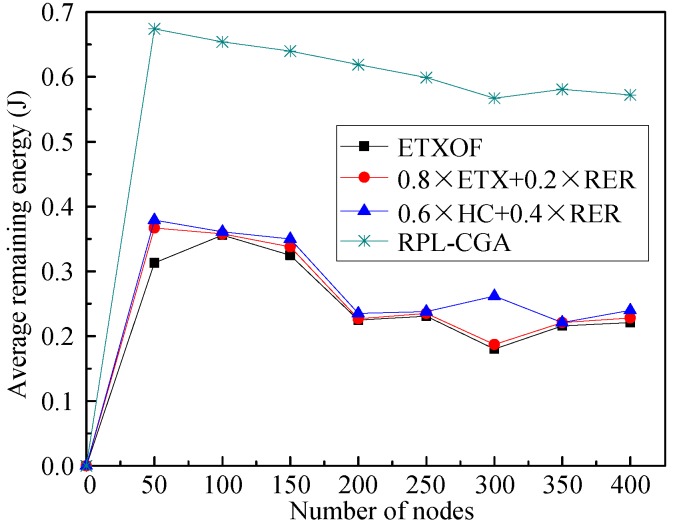
Average remaining energy.

**Figure 6:**
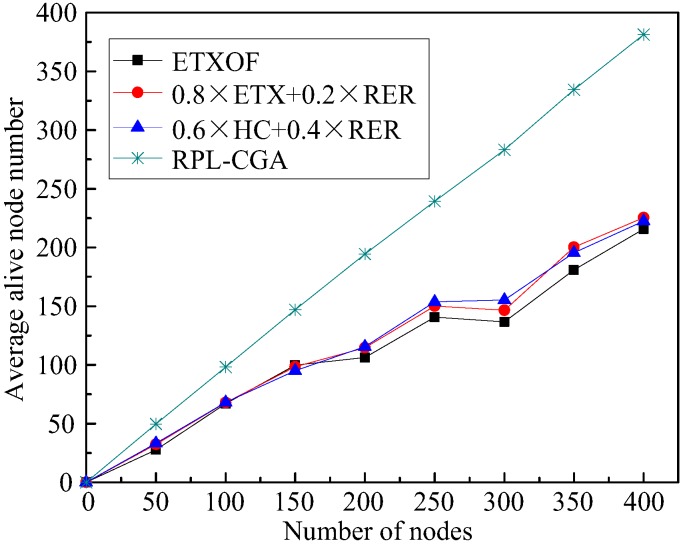
Average alive node number.

The average residual energy of nodes or average number of dead nodes in the network can clearly estimate the length of the network lifetime. Figure 5 and Figure 6 show RPL-CGA, ETXOF, 0.8 × ETX + 0.2 × RER, and 0.6 × HC + 0.4 × RER of these two statistical experimental indicators under several different node number circumstances. It can be seen that the statistical experimental indicators of ETXOF, 0.8 × ETX + 0.2 × RER, and 0.6 × HC + 0.4× RER are much lower than that of RPL-CGA at different node density circumstances. Therefore, by employing the chaotic genetic algorithm to determine the weighting factors of QL, EED, RER, HC, and ETX, RPL-CGA can prolong network lifetime significantly.

(4) Average hop count

**Figure 7:**
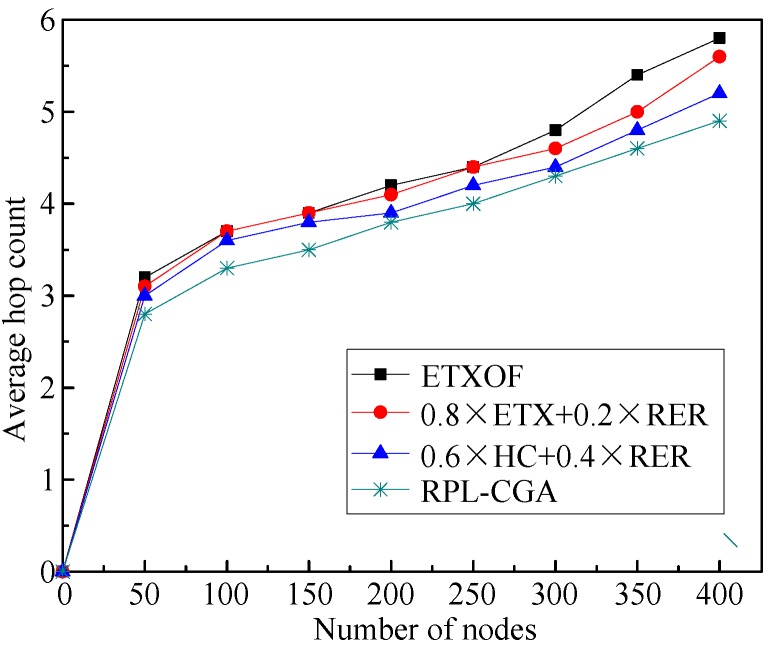
Average hop count.

The average hop count of RPL-CGA, ETXOF, 0.8 × ETX + 0.2 × RER, and 0.6 × HC + 0.4 × RER under several different node number circumstances are illustrated in Figure 7. The average hop count of RPL-CGA, ETXOF, 0.8 × ETX + 0.2 × RER, and 0.6 × HC + 0.4 × RER are almost the same at small node density, because the low node number in network restricts the choice of preferred parent. But with the increase of node number, the choice of a preferred parent is gradually unrestricted. Therefore, the hop count of ETXOF, 0.8 × ETX + 0.2 × RER, and 0.6 × HC + 0.4 × RER are much higher than that of RPL-CGA. Moreover, RPL-CGA can decrease hops from non-nodes to root dramatically.

(5) Average frequencies of preferred parent changing

**Figure 8:**
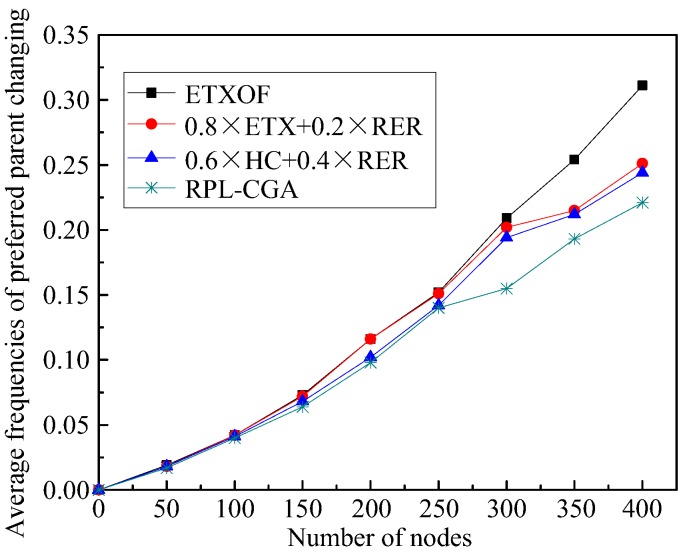
Average frequencies of preferred parent changing.

Average frequencies of preferred parent changing represent the stability of network topology. Figure 8 shows this statistical experimental indicator of RPL-CGA, ETXOF, 0.8 × ETX + 0.2 × RER, and 0.6 × HC + 0.4 × RER under several different node number circumstances. The average frequencies of preferred parent changing of ETXOF, RPL-CGA, 0.8 × ETX + 0.2 × RER, and 0.6 × HC + 0.4 × RER are not much different. So, on the one hand, RPL-CGA can significantly optimize several aspects of network performance; on the other, it can also guarantee the stability of network’s topology.

(6) Weighting factors (*a*_1_, *a*_2_, *a*_3_, *a*_4_, *a*_5_)

**Figure 9:**
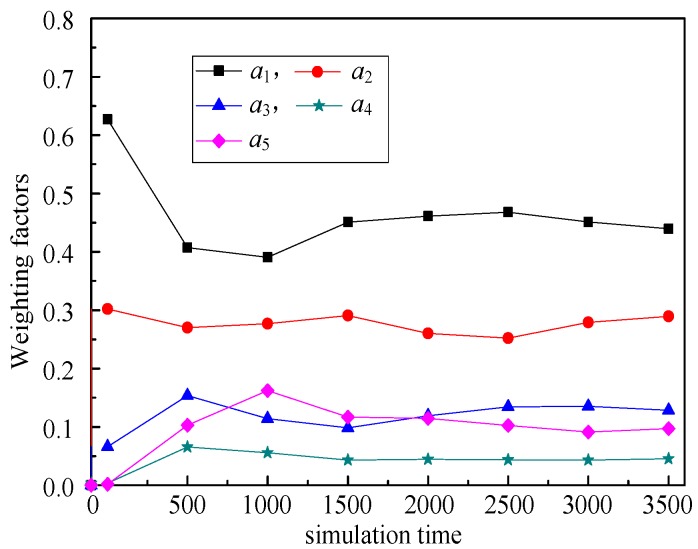
Relationship between weighting factors and simulation time: node 2.

**Figure 10:**
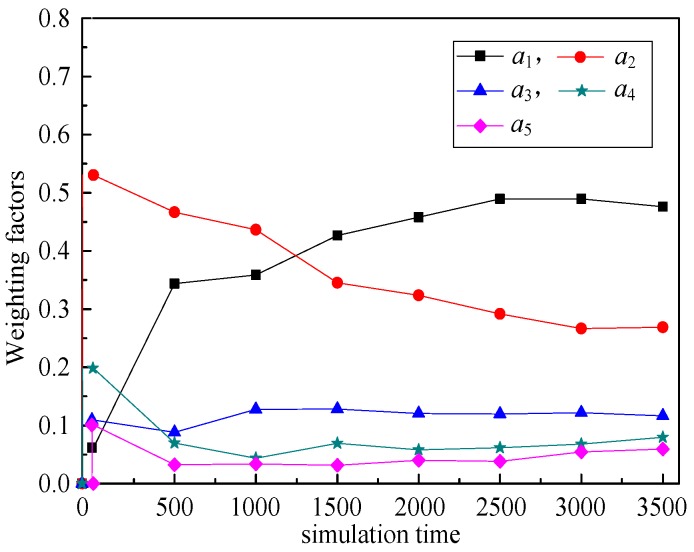
Relationship between weighting factors and simulation time: node 38.

**Figure 11:**
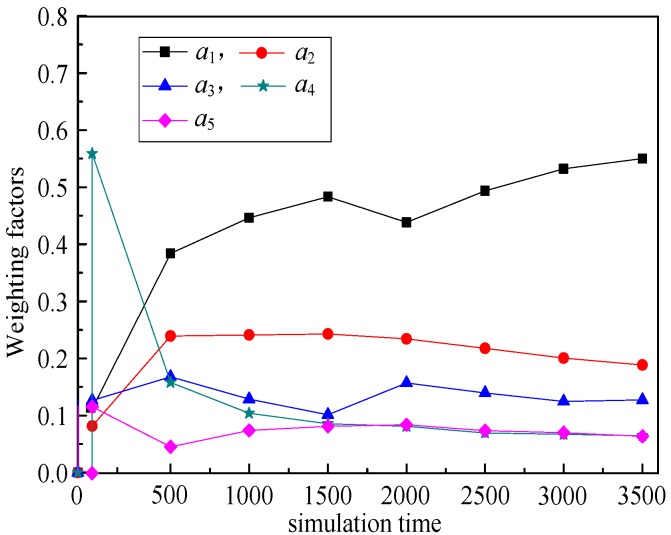
Relationship between weighting factors and simulation time: node 100.

**Figure 12:**
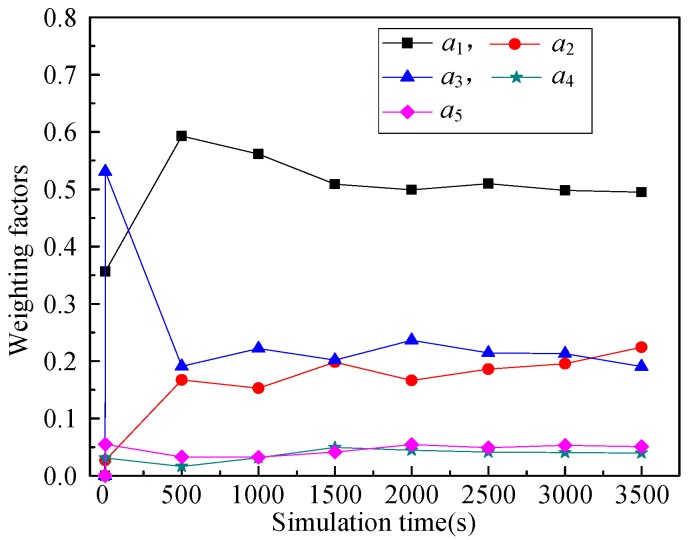
Relationship between weighting factors and simulation time: node 319.

Figure 9, Figure 10, Figure 11, Figure 12 illustrate the relationship between weighting factors (*a*_1_, *a*_2_, *a*_3_, *a*_4_, and *a*_5_) and simulation time respectively. We randomly selected nodes 2 (corresponding to Figure 9), 38 (corresponding to Figure 10), 100 (corresponding to Figure 11), and 319 (corresponding to Figure 12) to show the relationship between weighting factors and simulation time. It’s obvious that RPL-CGA can dynamically adjust the values of *a*_1_, *a*_2_, *a*_3_, *a*_4_, and *a*_5_ according to network changes, choose the optimum parents as preferred parents, and improve network performance effectively.

## 5. Discussion

Before concluding this paper, we discuss several relevant issues in our newly-proposed RPL-CGA. RPL-CGA proved its efficiency, and that its method of dealing with the problem of “subjectiveness” when using the additive metric approach was correct. But some parameters used in CGA, such as the value of α in Equation (15), cross probability *P_c_*, and mutation probability *P_m_*, are still not determined objectively. The reasonable parameter ranges and values used in CGA can only be determined by multiple experimental calculations, since currently, there is no theoretical basis. In this paper, the values of several parameters used in CGA are comprehensively determined by research literature and experimental calculations. Therefore, the subjective determination of these parameters has no significant influence on the network performance; instead, the network performance can be significantly improved, and is superior to the existing relevant algorithm.

## 6. Conclusions

In conclusion, RPL-CGA can comprehensively assess the five proposed routing metrics (QL, EED, RER, HC, and ETX) and combine these metrics according to Equation (9). Meanwhile, their respective weighting factors are determined by a chaotic genetic algorithm. Then, the selection of preferred parent can based on the minimum value of Equation (19). Finally, performance evaluation proves the effectiveness and reliability of RPL-CGA. Meanwhile, RPL-CGA is significantly superior to other improvements of RPL such as ETXOF, 0.8 × ETX + 0.2 × RER, and 0.6 × HC + 0.4 × RER in the aspect of average end-to-end delay, average success rate, etc. Further analysis should be focused on multiple instances of routing RPL to process different kinds of traffic flows in the network.

## Figures and Tables

**Table 1:** ICMPV6 control messages used in RPL.

ICMPV6 Control Messages	Functions
DAO (Destination Advertisement Object)	Transmitting destination address information and constructing upward routes
DIO (DODAG Information Object)	Containing some information that used to detect RPL Instance, obtain relevant configuration parameters, select candidate parent set, maintaining DODAG, etc.
DIS (DODAG Information Solicitation)	soliciting DIOs from neighbors in LLNs
DAO-ACK (Destination Advertisement Object Acknowledgement)	Informing DAO sender that DAO has been received

**Table 2:** Experiment parameters.

Parameter	Value
Network scenario (m^2^)	500 × 500
Dead node	Residual energy less than 5% of its initial energy
Maximum number of iteration *k*	100
Cross probability *P_c_*	0.75
Mutation probability *P_m_*	0.001
Population size *w*	100
Simulation time (s)	3000
Maximum queue length (packet number)	16
Minimum queue length (packet number)	0
Communication radius (m)	150
Node number	50, 100, 150, 200, 250, 300, 350, 400
Packet size (kbits)	0.1
Energy loss for relaying *y* bit message	*E*(*y*,*d*)

**Table 3:** The value of each parameter in *E*(*y*,*d*).

Parameter	Value
*E_elec_*	50 nJ/bit
*ε* *_amp_*	10 pJ/bit/m^2^
*ε* *_fs_*	0.0013 pJ/bit/m^4^
*d_0_*	87 m
*d*	communication distance
